# Dietary Assessment for Weight Management and Health Maintenance

**DOI:** 10.3390/nu15214610

**Published:** 2023-10-30

**Authors:** Cristina Reche-García, Juan José Hernández Morante, Juana M. Morillas-Ruiz

**Affiliations:** 1 Eating Disorders Research Unit, Campus de Los Jerónimos, Universidad Católica de Murcia, Guadalupe, 30107 Murcia, Spain; creche@ucam.edu; 2 Faculty of Pharmacy and Nutrition, Campus de Los Jerónimos, Universidad Católica de Murcia, Guadalupe, 30107 Murcia, Spain

An adequate dietary assessment is essential for improving the eating habits of the population and preventing health problems such as obesity and cardiovascular diseases. Dietary assessment has emerged as a pivotal factor in contemporary health paradigms [1]. The interplay between nutrition, weight management, and overall health has never been more profound. In an era where obesity and diet-related diseases are reaching alarming proportions [2], understanding the impact of dietary choices on individual health outcomes has become imperative.

There are various methods available for evaluating diets in the context of weight control. These methods are necessary to detect nutrition-related disorders or conditions, to characterize eating behaviors or patterns, and to compare nutritional interventions and verify their effectiveness. The evaluation of diet patterns is also essential to provide evidence in diet-based weight management programs or be aware of the dietary knowledge of specific populations [3].

Dietary assessment is not merely a matter of counting calories; it is the science of deciphering the intricate relationship between dietary patterns, nutrient intake, and health maintenance. In this editorial, we aim to gain a global perspective of the multifaceted landscape of dietary assessment, exploring its indispensable role in the context of weight management and health preservation. Throughout this Special Issue, we delve into three distinct topics of great interest: the relevance of dietary fats in health maintenance, the evaluation of emotional and mindful eating, and the development of classic methods, like the food frequency questionnaire, for specific populations, such as disadvantaged families.

Addressing a topic as broad as dietary assessment for weight management and health maintenance is difficult for a single Special Issue, although in general, we can affirm that several major topics on this area have been covered. One of these topics focused on the relevance of dietary fat and other bioactive components on health. There is a large quantity of studies that have previously evaluated the impact of dietary fat on human health [4,5,6]; however, the works of this issue confirm that there is still much to learn about the effect of consuming fat on health. The work of Delgado-Alarcón also demonstrated the relevance of breakfast on human health maintenance [7]. Concretely, this work highlighted the fact that a single modification of fat eaten at breakfast was enough to improve the inflammation profile of women. This randomized trial evaluated the influence of monounsaturated fatty acids (MUFAs), polyunsaturated fatty acids (PUFAs) and saturated fatty acids (SFAs) on a cluster of cytokines and other inflammation markers. The results obtained by Delgado-Alarcón et al. demonstrated that a MUFA-enriched breakfast was the best option for decreasing the synthesis of these markers, an effect that was especially evident for interleukin-6 (Il-6) and vascular/endothelial growth factor (VEGF) [7]. The other work that evaluated the effect of fat on human health was the study conducted by Portela et al. [8]. In this work, other topics of great interest, like human microbiomes, were studied. These authors studied the influence of omega-3 fatty acids (PUFAs) on gut microbiota, and they concluded that the administration of omega-3 fatty acids is able to make specific changes in microbiota, which, in turn, modulated the immune metabolic response of adipose tissue [8]. This work provides more information about the possible mechanisms of action of omega-3 fatty acids, which have been previously described as anti-inflammatory, hypocholesterolemic, and protective against cardiovascular events [9,10]. Not only fat and other nutrients, but also other non-nutritive bioactive components are of great relevance for weight management and health maintenance. We consider that this issue deserves its own Special Issue. Nevertheless, In-Seon et al. have described, through a systematic review, the benefits of *Laminaria japonica* (LJP) on metabolic syndrome [11], describing the effects of LJP on specific targets such as AMP-activated protein kinase, and decreases in acetyl-CoA carboxylase.

At present, the evaluation of the diet must go beyond simply evaluating energy and nutrient content. Several aspects like food addiction, hunger/satiety rhythms, food preferences, etc., must be considered. In this regard, two works have studied the assessment of emotional eating and mindful eating. Emotional eating is linked to stress, anxiety and other psychological traits. Previous works have described that emotions influence weight stability; therefore, it is necessary to consider this issue for a holistic dietary assessment. The work of Putnoky et al. confirmed the validity and reliability of the Romanian version of the Emotional Eater Questionnaire [12]. Interestingly, these authors employed the mindful eating questionnaire (MEQ) to evaluate concurrent validity. Precisely, Wang et al. also developed the Chinese version of the MEQ [13]. Mindful eating is the act of eating consciously, emphasizing physical and emotional sensations rather than judgment. Because people are less resistant to natural food cravings compared to dieting, mindful eating is considered an effective intervention to help people who are overweight lose weight safely. Previous works have described that mindful eating may serve as a prevention factor for weight maintenance. Therefore, this adaptation may be of great use for the Chinese population [13]. An interesting aspect of these studies is the need to continue adapting these questionnaires to different languages and contexts. We encourage researchers to advance these adaptations in order to include these measures in daily practice.

Finally, the present issue has included several reports which can advance our knowledge of classical methods for dietary assessments. Anthropometrical measurements have been a cornerstone in the evaluation of body composition, which is invaluable to confirm the effectiveness of dietary interventions. In this regard, the work of Esparza-Ros et al. described the validity of four different skinfold calipers and established the differences between them in a healthy young adult population [14]. This study highlights the need for accuracy and consistency when taking these anthropometric measurements, since, for example, the use of different calipers in the same study can increase the risk of systematic errors in anthropometric studies [14]. Nevertheless, the use of anthropometric measurements is not always feasible. For instance, in children, the reliability of skinfolds limits the applicability of these techniques. In this issue, we have examined the utility of sonographic markers for assessing the early markers of cardiovascular impairment [15]. In our view, these data hold significant importance for the prevention of obesity-related diseases in children. As in adults, childhood obesity is associated with multiple metabolic disorders. However, little information is available on complex obesity-related complications such as hepatic steatosis or endothelial dysfunction early in life. In this paper, we confirmed that sonographic markers are adequate for determining liver and carotid intima-media thickness (cIMT) parameters in children [15]. If we consider that these techniques are non-invasive and relatively accessible from an economic point of view, their use can help not only to prevent alterations, but also to develop dietary interventions aimed at reducing cardiovascular risks.

Several adaptations of food questionnaires were also developed. Although the food frequency questionnaire (FFQ) can be considered an outdated procedure, it is necessary to adapt this technique to specific groups, as carried out in this issue. Chan et al. have developed a short version of the FFQ for disadvantaged families [16]. People at a lower socioeconomic level are more susceptible to dietary difficulties since the diet quality of these families is usually inadequate. In addition, the work of Conti et al. also developed a FFQ for other specific collectives, such as Tanzanian childbearing women [17]. Again, we consider it indispensable to validate these specific tools for a better understanding of diet characteristics. On the other hand, although understanding the intake of certain specific nutrients is important, today it is well established that it is better to understand the combination of these nutrients and their influence on health. For this, the concept of the dietary index has been developed, such as the healthy eating index, diet quality index, etc. In this Special Issue, Jannasch et al. developed a new diet score, based on the German dietary guideline, that will be of great interest as a tool to investigate dietary intake in patients with chronic diseases [18]. Undoubtedly, new research is needed to confirm the validity of this new index for large population studies.

To summarize, we are thankful to the publisher for trusting us to edit this Special Issue. Although works of great interest have been covered, there are important aspects that have also been left unaddressed. In future work, the importance of new high-performance computing techniques, together with machine learning procedures, will bring us closer to a more personalized precision diet [19]. We would also have liked to comment on the relevance of omics techniques in future dietary assessments. Thanks to metagenomics [20] or epigenomics [21] techniques, we may be able to describe the effects of dietary components on weight management and health maintenance more precisely.
